# Impact of Brodalumab on Serum Levels of IL-6, IL-17A, IFN-α, IFN-γ, and TNF-α in Patients with Psoriasis Who Failed Treatment with TNF-α Inhibitors

**DOI:** 10.3390/ijms27010458

**Published:** 2026-01-01

**Authors:** Lucia Medjedovic, Admir Vižlin, Ylva Andersch Björkman, Anna-Maj Albertsson, Sukanya Raghavan, Martin Gillstedt, Amra Osmancevic

**Affiliations:** 1 Department of Dermatology and Venereology, Institute of Clinical Sciences, Sahlgrenska Academy, University of Gothenburg, 413 45 Gothenburg, Sweden; lucia.medjedovic@gmail.com (L.M.); admir.vizlin@gmail.com (A.V.);; 2 Department of Dermatology and Venereology, Sahlgrenska University Hospital, Region Västra Götaland, 413 45 Gothenburg, Sweden; 3 Department of Clinical Immunology and Transfusion Medicine, Sahlgrenska University Hospital, Region Västra Götaland, 413 45 Gothenburg, Sweden

**Keywords:** psoriasis, brodalumab, IL-17 inhibitor, TNF-α inhibitor, cytokines

## Abstract

Psoriasis is a chronic, immune-mediated inflammatory skin disorder that significantly impacts patients’ quality of life. While TNF-α inhibitors are frequently used to treat moderate-to-severe cases, not all patients respond adequately. Brodalumab, a monoclonal antibody targeting the IL-17 receptor A, has emerged as an alternative for individuals unresponsive to prior therapies. This prospective study investigated the effects of brodalumab on serum cytokine levels—specifically IL-6, IL-17A, IFN-α, IFN-γ, and TNF-α—and their correlation with disease severity as assessed by Psoriasis Area and Severity Index (PASI). Eighteen patients with moderate-to-severe psoriasis who were unresponsive to TNF-α inhibitors received brodalumab for 12 weeks. Cytokine concentrations were measured at baseline and week 12 using an automated immunoassay (ELLA), and clinical outcomes were evaluated using PASI. The results showed a significant increase in IL-17A levels, while changes in IL-6, IFN-α, IFN-γ, and TNF-α did not reach statistical significance. No significant correlations were found between changes in cytokine levels and PASI improvement. However, the small number of available serum samples at week 12 (*n* = 11) limited the statistical power to detect treatment-related changes in cytokine levels. These findings suggest that while brodalumab influences specific immune markers, the clinical response may not be directly reflected by serum cytokine levels. This highlights the multifactorial nature of psoriasis pathogenesis and underscores the need for further studies to clarify the role of cytokine biomarkers in treatment response.

## 1. Introduction

Psoriasis is a widespread chronic and immune-mediated skin condition that affects both men and women globally and can manifest at any age. The most common form, psoriasis vulgaris, is characterized by well-defined, scaly plaques that appear salmon-pink on white skin and gray on darker skin. Lesions are usually located on elbows, knees, the scalp, and the lower back. The reported prevalence of psoriasis varies widely, ranging from 0.09% in some regions to as high as 11.4% in others. In most developed nations, prevalence rates typically fall between 1.5% and 5%. Psoriasis is often associated with a considerable decline in quality of life and negative effects on psychosocial well-being, affecting the majority of patients [1].

The underlying cause of psoriasis is not fully understood, and there is currently no cure [2]. The disease is multifactorial, arising from a complex interaction between genetic and environmental factors [1]. Psoriasis is increasingly recognized not only as a skin condition but also as a systemic inflammatory disease. This systemic nature is reflected in its association with several comorbidities, including cardiovascular disease, metabolic syndrome, mental health disorders, and psoriatic arthritis (PsA), all of which contribute to higher morbidity and reduced quality of life [1,3].

At the cellular level, key immune cells include T-cells, dendritic cells, neutrophils, and keratinocytes. These cells communicate primarily through pro-inflammatory cytokines such as tumor necrosis factor (TNF)-α, interferon (IFN)-γ, interleukin (IL)-17, and IL-22, and through the activation of keratinocytes, which further release antimicrobial proteins, growth factors, and chemokines [1].

IFN-α is involved in the early formation of psoriatic plaques and, when released, leads to secretion of IL-12 and IL-23 [4]. It can also directly promote the activation and proliferation of T helper 1 (Th1) and Th17 cells [5,6]. IL-17A, commonly referred to as IL-17, is part of the IL-17 cytokine family (IL-17A–F) and is the most extensively studied member, with the strongest association to human health and disease [7]. IL-17A, mainly produced by Th17 cells, acts on keratinocytes, promoting their hyperproliferation and stimulating them to secrete chemokines that attract additional immune cells [5]. TNF-α is an important proinflammatory cytokine in psoriasis pathogenesis that is released by immune cells and keratinocytes [1,5]. TNF-α stimulates the release of additional pro-inflammatory cytokines and promotes immune cell recruitment to the skin, sustaining a chronic inflammatory environment. This cytokine also amplifies the effects of other pro-inflammatory mediators, such as IL-17 and IL-23, which together intensify keratinocyte proliferation and the formation of psoriatic plaques [1,4]. IL-6 is a pro-inflammatory cytokine released by keratinocytes in response to stimulation by TNF-α and IL-1. This cytokine not only supports the growth and differentiation of keratinocytes but also facilitates T-cell migration to the epidermis, contributing to psoriasis-related inflammation [8]. IFN-γ is primarily produced by Th1 cells and is released in response to cytokines like IL-12. Once released, IFN-γ enhances the inflammatory response in several ways, including improving antigen presentation, facilitating T-cell activation and differentiation, sensitizing keratinocytes, and enhancing their responsiveness to other pro-inflammatory cytokines. Furthermore, IFN-γ stimulates immune cells to release cytokines such as IL-1 and IL-23, which promote further T-cell differentiation, especially of IL-17-producing cells, integral to the psoriatic inflammatory response [5].

Two widely utilized clinical assessment tools for psoriasis are the Psoriasis Area and Severity Index (PASI) and the Dermatology Life Quality Index (DLQI) [2]. In Sweden, psoriasis severity is classified using a combination of Body Surface Area (BSA), PASI, and DLQI. Mild disease is defined as BSA < 3%, PASI < 3, and DLQI ≤ 5; moderate-to-severe disease as BSA 3–10%, PASI 3–10, and DLQI 6–10; and severe disease as BSA > 10%, PASI > 10, and DLQI > 10 [9]. While PASI is widely used to assess disease severity, it has several limitations. The scale is non-linear, making interpretation challenging, and it is less sensitive to changes at lower scores while becoming redundant at higher scores. Moreover, PASI does not account for comorbidities or involvement of specific body areas—such as the hands, nails, feet, face, and genital region—that can cause substantial impairment despite not meeting criteria for severe disease [10,11].

Over the past decade, the IL-23/Th17 axis has been recognized as a central driver of plaque psoriasis, leading to the development of IL-17 inhibitors as targeted therapies. These drugs work by either blocking the IL-17 ligand or its receptor. This stops the inflammatory pathways that IL-23/Th17 signaling causes [12]. Brodalumab, an IL-17 receptor A inhibitor, has demonstrated strong clinical efficacy in patients with psoriasis [4,13]. However, the factors determining why some patients respond better than others are not fully understood. Given the high costs of biologic therapies and the significant impact of the disease on affected patients, it is essential to ensure that treatment is directed toward those most likely to benefit. Previous studies have investigated whether specific cytokine profiles in patients with psoriasis may predict treatment response to biologic agents [14,15,16]. Other studies have explored associations between genetic polymorphisms and treatment response to biologics in psoriasis [16,17,18]. However, there are very few—if any—studies that have examined genetic polymorphisms or serum cytokine levels before and after treatment with brodalumab, leaving an important gap in our understanding of biomarkers for treatment response.

In this study, we investigated serum levels of IL-6, IL-17A, IFN-α, IFN-γ, and TNF-α in patients with psoriasis before and after treatment with brodalumab, all of whom had previously failed therapy with TNF-α inhibitors. We also examined whether changes in serum cytokine levels correlated with clinical outcomes, as assessed by PASI.

## 2. Results

### 2.1. Patient Characteristics

Baseline demographics, clinical characteristics, and comorbidities were derived from data collected in our previously conducted phase 4 study and are summarized in Table 1 for descriptive purposes [19]. The majority of participants were male (*n* = 14), with a mean age of 49.6 years and a mean Body Mass Index (BMI) of 30. Most were non-smokers (*n* = 17). Medical histories were available for 14 of the 20 patients, as records for the remaining six were inaccessible. Of these 14, five had medically controlled comorbidities, including hypertension, diabetes, hyperlipidemia, and obesity. The PASI and DLQI values reported in Table 1 (mean ± SD) reflect screening assessments of all 20 patients included in our previous phase 4 study, performed prior to allocation to TNF-α inhibitor treatment or direct initiation of brodalumab.

### 2.2. Changes in PASI and DLQI

At baseline prior to brodalumab initiation, the mean PASI and DLQI scores for all 20 patients were 8.8 and 10.8, respectively. Seventeen patients completed all study visits, with slightly lower baseline mean scores of 8.7 for PASI and 10.1 for DLQI in this subgroup. In these patients, the mean PASI score decreased significantly from 8.7 to 2.5 after 12 weeks of brodalumab treatment (*p* < 0.0001), while the mean DLQI score decreased from 10.1 to 4.1 (*p* = 0.004). However, three patients did not meet the Swedish guidelines for treatment response after 12 weeks: two with PASI > 7 and one with PASI 6.4 and DLQI > 5.

### 2.3. Changes in Cytokine Levels

Of the 20 patients included in the study, serum samples for cytokine analysis were available at baseline for 18 patients; samples from two patients were not collected despite being scheduled according to study records. At week 12, serum samples were available from only 11 patients, as planned follow-up sampling was not completed for the remaining six patients. All 11 week-12 serum samples corresponded to the same patients for whom baseline samples were available, allowing paired comparisons to be performed using the Wilcoxon signed-rank test. The analysis of serum cytokine levels before and after 12 weeks of brodalumab treatment revealed varied changes across different cytokines (Table 2). IL-17A levels increased significantly, with a mean change of +7.016 pg/mL (*p* = 0.002, range: −0.206–15.168, SD: 4.656 pg/mL), indicating a substantial response. TNF-α levels exhibited a mean decrease of −14.85 pg/mL, nearing statistical significance (*p* = 0.067, range: −44.8–2.46 pg/mL, SD: 18.343 pg/mL), suggesting a potential trend toward reduced inflammation. IL-6 levels showed a mean increase of +0.356 pg/mL, though this change was not statistically significant (*p* = 0.206, range: −1.24–2.22 pg/mL, SD: 0.939 pg/mL). IFN-α levels decreased slightly, with a mean change of –0.264 pg/mL, which was also not statistically significant (*p* = 0.206, range: −1.278–0.451 pg/mL, SD: 0.566 pg/mL). Lastly, IFN-γ levels showed a mean increase of +0.119 pg/mL, which was not statistically significant (*p* = 0.520, range: −1.227–2.14 pg/mL, SD: 1.049 pg/mL). Concentrations of IL-17A and IFN-α were generally low, with most IFN-α values and a substantial proportion of IL-17A values falling below the assay’s validated lower limit of quantification (LLOQ). These values were detectable but considered semi-quantitative, and were retained to allow assessment of relative changes over time.

### 2.4. Correlations Between Changes in PASI and Cytokine Levels

Spearman’s correlation analysis revealed no statistically significant associations between changes in PASI scores and cytokine levels after 12 weeks of treatment (Table 3). Correlation coefficients are presented together with their corresponding 95% confidence intervals (CI). A moderate negative correlation was observed between PASI change and IFN-γ change (*ρ* = −0.51, *p* = 0.11); however, this association did not reach statistical significance after adjustment for multiple testing. The remaining cytokines, including IL-17A (*ρ* = 0.059), IL-6 (*ρ* = 0.14), TNF-α (*ρ* = −0.36), and IFN-α (*ρ* = 0.41), demonstrated weak to moderate correlations with PASI change, but none were statistically significant after adjustment (adjusted *p* = 1 for all).

**Figure 1:**
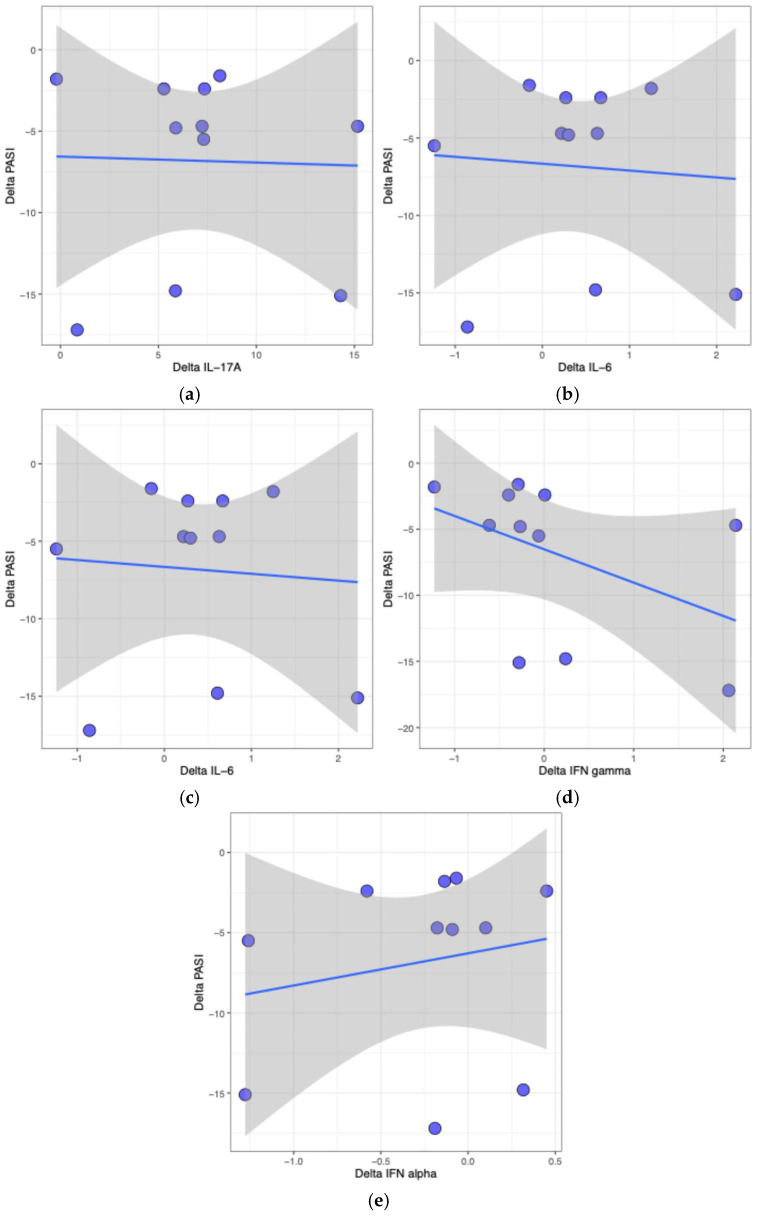
Scatter plots visualizing the relationship between changes in PASI score (ΔPASI) and changes in cytokine concentrations (Δcytokine) for individual patients. Each dot represents a single participant. Panels show the following cytokines: (**a**) IL-17A, (**b**) IL-6, (**c**) TNF-α, (**d**) IFN-γ, and (**e**) IFN-α. The *x*-axis denotes the change in cytokine level, and the *y*-axis represents the corresponding change in PASI. The blue line indicates the fitted linear regression, and the shaded grey area represents the 95% confidence interval around the estimated regression line. The distribution of points illustrates the range of observed changes across patients. These plots provide a visual representation of individual data points, allowing assessment of the spread and overlap of changes in cytokine levels and clinical outcomes. Correlations between ΔPASI and Δcytokine were not statistically significant (*p* > 0.05).

To complement the correlation analysis, scatter plots were generated to visualize the relationships between changes in PASI score (ΔPASI) and changes in cytokine concentrations (Δcytokine) for individual patients (Figure 1). Each point represents a patient, providing a visual overview of the variation in responses and allowing evaluation of how changes in cytokine levels relate to clinical outcomes. The plots showed a wide dispersion of data points and no clear trends. This is consistent with the Spearman analysis, which revealed no statistically significant correlations.

## 3. Discussion

This study revealed significant changes in cytokine levels after 12 weeks of treatment with brodalumab in patients who had previously failed therapy with TNF-α inhibitors. Among the analyzed cytokines, IL-17A levels showed a statistically significant increase (*p* = 0.002), consistent with the mechanism of action of brodalumab as an IL-17 receptor A blocker. In contrast, changes in IL-6, TNF-α, IFN-γ, and IFN-α were not statistically significant. However, TNF-α levels showed a trend toward reduction (*p* = 0.067), which may reflect prior exposure to TNF-α inhibitors before the initiation of brodalumab. These findings highlight the specificity of brodalumab’s action on IL-17 receptor A and its effects on IL-17A.

The observed increase in serum IL-17A levels aligns with the pharmacodynamics of brodalumab, which inhibits IL-17 signaling by blocking the IL-17 receptor A rather than neutralizing IL-17A directly. This receptor blockade prevents receptor-mediated degradation of IL-17A, resulting in its accumulation in circulation. Importantly, this rise in circulating IL-17A does not indicate heightened systemic inflammation but reflects the pharmacological consequence of unbound cytokines diffusing into the bloodstream [20].

Several studies have reported similar increases in IL-17A after brodalumab treatment [20,21,22]. However, Loft et al. found a contrasting pattern, with a significant decrease in IL-17A concentrations after 12 weeks. In the study conducted by Loft et al., prior treatment with one or more IL-17A inhibitors was an inclusion criterion, and patients were required to have achieved less than a 50% reduction in PASI or to have experienced loss of response. In contrast, our study excluded patients with any previous IL-17 inhibitor treatment. Furthermore, patients in the study by Loft et al. underwent a four-week washout period from all systemic psoriasis treatments prior to baseline [14], whereas no such washout was applied in our study. These methodological differences could partly explain why IL-17A levels decreased in their study while increasing in ours. Another possible reason for the discrepancy between their findings and ours is individual differences in baseline inflammatory activity, where patients with higher initial cytokine levels shift toward a more normalized state during treatment.

It should be noted that, overall, very few studies have investigated how cytokine levels change before and after brodalumab treatment in psoriasis patients. Consequently, the limited available studies cited in this article are used primarily for contextual comparison, regardless of differences in prior IL-17 inhibitor exposure, and direct extrapolation should be interpreted with caution. Similarly, while the observed increase in circulating IL-17A aligns with the expected pharmacology of IL-17RA blockade, no patient-level studies have directly demonstrated receptor-mediated cytokine accumulation in psoriasis. Therefore, interpretations regarding IL-17A dynamics are based on mechanistic reasoning rather than empirical patient data.

Loft et al. also reported that patients who responded to treatment within 12 weeks had significantly lower baseline levels of IL-6 and TNF-α compared to non-responders [14]. We did not perform a corresponding comparison since only three individuals were classified as non-responders, which limited the possibility of a meaningful analysis. Such an assessment might otherwise have provided additional insight into whether similar patterns were present in our cohort.

For the other cytokines analyzed, the absence of significant changes may suggest that the effects of brodalumab are primarily localized to the IL-17 pathway. Nevertheless, the near-significant reduction in TNF-α could indicate secondary immunological effects that merit further investigation. One possible explanation for the lack of a stronger reduction in TNF-α levels is prior TNF-α inhibitor therapy, which may have already lowered their TNF-α levels to an extent that limited further measurable decreases. This trend is noteworthy given the central role of TNF-α in inflammation, its established connection to the pathogenesis of psoriasis, and its relevance as a treatment target [1]. The absence of significant changes in IL-6, IFN-γ, and IFN-α may suggest that these cytokines are less directly affected by IL-17 receptor A blockade or that changes occur on a timescale longer than 12 weeks.

In this study, we explored potential associations between changes in PASI and serum cytokine levels. No statistically significant correlations were observed for any of the cytokines. A moderate negative correlation was noted between IFN-γ and ΔPASI (ρ = –0.51, *p* = 0.11), but this association did not reach statistical significance. These analyses should be interpreted with caution given the limited number of available samples at week 12, which constrained the ability to detect moderate correlations. A study by Navrazhina et al. reported that, in patients with hidradenitis suppurativa, higher baseline serum IL-17A levels were associated with greater reductions in inflammatory cytokines in perilesional skin after 12 weeks of brodalumab treatment [22]. These findings suggest that elevated pretreatment IL-17A levels may predict a more favorable therapeutic response. Since hidradenitis suppurativa, like psoriasis, is a chronic inflammatory skin disease where IL-17 plays a central pathogenic role [1,23], these observations may also be relevant for anticipating treatment outcomes in psoriasis. These observations raise the possibility that baseline IL-17A levels could serve as a biomarker to guide patient selection for brodalumab treatment, although this requires confirmation in larger studies.

Several factors could contribute to the results in this study. First, comorbidities commonly associated with psoriasis, such as obesity, metabolic syndrome, and cardiovascular disease, are known to affect systemic cytokine levels and may have confounded the observed correlations [24]. At least five of the patients in the study had either hypertension, diabetes, hyperlipidemia, or obesity, which could have influenced cytokine levels in this study. However, it should be emphasized that these conditions were medically treated and well-controlled. Moreover, prior and concurrent therapies, such as TNF-α inhibitors or methotrexate, might have lingering effects on cytokine dynamics, further complicating the interpretation of changes in cytokine levels. It is also worth considering the timing of cytokine measurements. Cytokine levels fluctuate during treatment, and measuring only at baseline and 12 weeks may fail to capture transient changes or peak effects. Furthermore, variability in patient response to brodalumab, including differences in baseline inflammation and receptor saturation, might obscure clear correlations. 

Despite these limitations, the study utilized a robust and reliable immunoassay platform (ELLA) with a high sensitivity and reproducibility (CV < 10%), ensuring accurate cytokine measurements. However, the limited availability of serum samples at week 12 (*n* = 11) may have reduced the statistical power to detect treatment-related changes, particularly for cytokines with smaller effect sizes, such as TNF-α, IFN-γ, and IL-6.

A key limitation of this study is the absence of a control or placebo group. Natural fluctuations in serum cytokine levels can occur in patients with psoriasis, and without a comparator, it is not possible to definitively attribute observed changes to brodalumab treatment. Consequently, while the observed increase in IL-17A aligns with the known pharmacological effects of brodalumab, caution is warranted in interpreting this change as solely treatment-induced. Future studies incorporating both placebo and untreated control groups would provide a more robust framework to distinguish true treatment effects from background biological variability.

Interpretation of IL-17A and IFN-α concentrations is limited by the fact that most IFN-α values and a substantial proportion of IL-17A values were below the assay’s validated lower limit of quantification. These measurements should therefore be considered semi-quantitative, and the findings are best interpreted in terms of relative changes rather than absolute serum levels.

Future research should aim to expand the sample size to enhance the statistical power and allow for subgroup analyses, such as those based on comorbidities. Longitudinal studies that include additional time points, both earlier and later than week 12, would help elucidate the temporal dynamics of cytokine changes during and after treatment. Furthermore, exploring the relationship between changes in serum cytokine levels and clinical outcomes, such as PASI and DLQI, could clarify the role of cytokines as potential biomarkers for treatment efficacy. Advanced statistical approaches, such as multivariate modeling, could be applied to identify specific combinations or patterns of cytokines that are predictive of clinical response.

Finally, integrating local tissue analyses, such as cytokine expression in psoriatic lesions, with systemic data could provide a deeper understanding of how brodalumab modulates both local and systemic inflammation. Such studies would be valuable for tailoring treatment strategies and understanding the broader implications of IL-17 receptor inhibition in psoriasis and related conditions.

## 4. Materials and Methods

This prospective observational study was conducted at the Department of Dermatology and Venereology, Sahlgrenska University Hospital, Gothenburg, Sweden. The study population, serum samples, clinical assessment scores (PASI and DLQI), and patient characteristics were derived from our previously conducted phase 4, open-label, randomized, parallel-group, multicenter study evaluating the efficacy of brodalumab in patients with psoriasis [19]. The present analysis constitutes an ancillary study, using these previously collected serum samples to measure cytokine levels and explore their relationship to clinical outcomes, which were not examined in the original trial.

### 4.1. Patient Recruitment

Patients were recruited consecutively from dermatology clinics in Sweden, where biologic treatments are commonly prescribed. Recruitment occurred during routine visits to outpatient dermatology clinics at the Departments of Dermatology and Venereology at University Hospitals in Gothenburg and Malmö, as well as dermatology departments at hospitals in Norrköping, Jönköping, and Eskilstuna. The recruitment period lasted from 6 December 2018 to 29 January 2020.

### 4.2. Inclusion and Exclusion Criteria

Eligible participants were adults (≥18 years) with a history of at least four months of treatment using TNF-α inhibitors and either PASI > 7 or PASI 3–7 with DLQI > 5. Exclusion criteria included the presence of any active infection requiring systemic treatment, uncontrolled systemic diseases (renal, cardiac, hepatic, or metabolic), a history of IBD, prior IL-17 inhibitor treatment, or pregnancy. Proficiency in the Swedish language and the absence of significant mental or cognitive impairments were further eligibility requirements. Written informed consent was obtained prior to inclusion. Patients were to be withdrawn from the study if severe adverse events or conditions interfered with brodalumab treatment.

### 4.3. Participant Overview and Dosing Regimen

Participants received brodalumab 210 mg at weeks 0, 1, and 2, followed by 210 mg every two weeks for a total of 12 weeks. Of 22 initially invited patients, 20 met inclusion criteria (14 men, 6 women), and 17 completed all study visits; all but one had a PASI ≥ 3 at baseline. Reasons for withdrawal included joint pain (*n* = 1), travel-related challenges (*n* = 1), and non-specific stomach issues during TNF-α treatment (*n* = 1) [19].

### 4.4. Serum Collection and Analysis

Serum samples were collected at baseline (week 0) and week 12. Samples were stored at −80 °C and analyzed using the ELLA automated immunoassay platform (ProteinSimple/Bio-Techne, Minneapolis, MN, USA) according to the manufacturer’s protocol. The validated quantifiable ranges were the following: TNF-α 0.3–1160 pg/mL, IFN-α 1.46–13,870 pg/mL, IFN-γ 0.17–4000 pg/mL, IL-6 0.28–2652 pg/mL, and IL-17A 1.05–10,000 pg/mL. The ELLA system demonstrates high precision, with intra-assay and inter-assay coefficients of variation (CV) typically below 10%, ensuring consistent and reliable cytokine measurements.

### 4.5. Clinical Assessments

Disease severity and quality of life were evaluated using PASI and DLQI at baseline and week 12. Medical histories, including comorbidities such as hypertension, diabetes, hyperlipidemia, and obesity, were reviewed from patient records. Detailed patient characteristics and baseline scores are presented in the Section 2 [19].

### 4.6. Statistical Analysis

Data were analyzed using R version 3.5.3 (The R Foundation for Statistical Computing, Vienna, Austria). Paired data comparisons were conducted with the Wilcoxon signed-rank test, while Spearman’s correlation test was used for testing correlations. The Benjamini-Yekutieli method was used to adjust for multiple tests. All tests were two-sided, and *p*-values < 0.05 were considered statistically significant.

## 5. Conclusions

This study highlights the significant impact of brodalumab treatment on cytokine dynamics, particularly the marked increase in IL-17A levels after 12 weeks. The findings align with the expected pharmacological mechanism of IL-17 receptor blockade, which leads to an accumulation of IL-17A in serum while inhibiting its inflammatory effects. Despite the lack of statistically significant changes in other cytokines, such as IL-6, TNF-α, IFN-γ, and IFN-α, the observed trends warrant further investigation to better understand the broader immunological effects of brodalumab. The results provide valuable insights into the systemic effects of IL-17 receptor inhibition in patients with psoriasis. However, the study is limited by the relatively small number of serum samples, which may have reduced the statistical power to detect subtle cytokine changes, highlighting the need for larger studies to confirm these findings.

## Figures and Tables

**Table 1:** Baseline characteristics of patients at study entry.

Variable	Value Total (*n* = 20)
Age, years	
Mean (SD)	49.6 (11.3)
Median	50.5
Min, Max	30, 70
Sex *n* (%)	
Female	6 (30)
Male	14 (70)
Ethnicity *n* (%)	
Hispanic or Latino	1 (5.0)
Not Hispanic or Latino	19 (95)
BMI, mean (SD)	30 (4.3)
Tobacco (*n*)	
Smokers	3
Age at first diagnosis of psoriasis, years	
Mean (SD)	21.8 (10.1)
Median	17.5
Min, Max	11, 44
Involvement of scalp (%)	
Yes	13 (65)
No	7 (35)
Involvement of nails (%)	
Yes	8 (40)
No	12 (60)
Involvement of psoriatic arthritis *n* (%)	
Yes	3 (15)
No	17 (85)
PASI, mean (SD)	9.3 (3.5)
DLQI, mean (SD)	10.3 (7.2)

Abbreviations: BMI, body mass index; DLQI, Dermatology Life Quality Index; PASI, Psoriasis Area and Severity Index.

**Table 2:** Cytokine levels measured at baseline (week 0) and after treatment (week 12).

Cytokine	Week 0	Week 12	*p*-Value
Patients	(*n* = 18)	(*n* = 11)	
IFN-α pg/mL, mean (min–max)	1.55 (0.08–12.30)	0.74 (0.17–2.84)	0.206
IL-6 pg/mL, mean (min–max)	3.99 (1.01–18.00)	3.45 (1.75–8.10)	0.206
IFN-γ pg/mL, mean (min–max)	1.04 (0.20–2.17)	1.08 (0.21–3.15)	0.520
IL-17A pg/mL, mean (min–max)	0.59 (0.00–2.15)	7.38 (0.73–15.20)	**0.002**
TNF-α pg/mL, mean (min–max)	31.07 (8.14–66.90)	12.03 (9.90–15.00)	0.067

Abbreviations: IFN, interferon; IL, interleukin; *n*, number; TNF, tumor necrosis factor. *p*-values < 0.05 are marked in bold.

**Table 3:** Correlations between cytokine levels and PASI scores at baseline (week 0) and after treatment (12 weeks).

Week 0	Spearman’s *ρ*	95% CI for Spearman’s *ρ*	*p*-Value	*p*-Value Adjusted
IFN-α	0.32	−0.16–0.72	0.2	1
IL-6	0.25	−0.32–0.71	0.31	1
IFN-γ	−0.01	−0.55–0.52	0.98	1
IL-17A	0.2	−0.35–0.69	0.43	1
TNF-α	−0.32	−0.67–0.17	0.2	1
**Week 12**				
IFN-α	0.41	−0.29–0.83	0.21	1
IL-6	0.14	−0.66–0.83	0.69	1
IFN-γ	−0.51	−0.86–0.07	0.11	1
IL-17A	0.059	−0.64–0.74	0.86	1
TNF-α	−0.36	−0.82–0.31	0.28	1

Spearman’s correlations between cytokine levels and PASI scores at baseline (week 0) and over 12 weeks of treatment. Correlation coefficients (*ρ*) are shown with 95% confidence intervals (CI) and *p*-values adjusted for multiple comparisons.

## Data Availability

Data available on request due to restrictions. Data from the study on the group level are publicly available. However, individual patient data are protected due to ethical reasons.

## References

[1] Griffiths C.E.M., Armstrong A.W., Gudjonsson J.E., Barker J. (2021). Psoriasis. Lancet.

[2] Michalek I., Loring B., John S. (2016). WHO Global Report on Psoriasis.

[3] Boehncke W.H., Schön M.P. (2015). Psoriasis. Lancet.

[4] Rendon A., Schäkel K. (2019). Psoriasis Pathogenesis and Treatment. Int. J. Mol. Sci..

[5] Sieminska I., Pieniawska M., Grzywa T.M. (2024). The Immunology of Psoriasis-Current Concepts in Pathogenesis. Clin. Rev. Allergy Immunol..

[6] Tohyama M., Yang L., Hanakawa Y., Dai X., Shirakata Y., Sayama K. (2012). IFN-α enhances IL-22 receptor expression in keratinocytes: A possible role in the development of psoriasis. J. Investig. Dermatol..

[7] McGeachy M.J., Cua D.J., Gaffen S.L. (2019). The IL-17 Family of Cytokines in Health and Disease. Immunity.

[8] Dowlatshahi E.A., van der Voort E.A., Arends L.R., Nijsten T. (2013). Markers of systemic inflammation in psoriasis: A systematic review and meta-analysis. Br. J. Dermatol..

[9] Duvetorp A., Osmancevic A., Stymne B., Enerbäck C., Lysell J., Sandberg S., Talme T., Särnhult T., Svensson Å. (2024). SSDV:s Behandlingsrekommendationer för Systemisk Behandling av Psoriasis (2024-12-15).

[10] Puzenat E., Bronsard V., Prey S., Gourraud P.A., Aractingi S., Bagot M., Cribier B., Joly P., Jullien D., Le Maitre M. (2010). What are the best outcome measures for assessing plaque psoriasis severity? A systematic review of the literature. J. Eur. Acad. Dermatol. Venereol..

[11] Golbari N.M., van der Walt J.M., Blauvelt A., Ryan C., van de Kerkhof P., Kimball A.B. (2021). Psoriasis severity: Commonly used clinical thresholds may not adequately convey patient impact. J. Eur. Acad. Dermatol. Venereol..

[12] Reid C., Griffiths C. (2020). Psoriasis and Treatment: Past, Present and Future Aspects. Acta Derm. Venereol..

[13] Armstrong A.W., Read C. (2020). Pathophysiology, Clinical Presentation, and Treatment of Psoriasis: A Review. JAMA.

[14] Loft N., Bregnhøj A., Fage S., Nielsen C.H., Enevold C., Zachariae C., Iversen L., Skov L. (2021). Effectiveness of brodalumab after previous treatment failure of interleukin-17A inhibitors in patients with psoriasis. Dermatol. Ther..

[15] Andersen C.S.B., Kvist-Hansen A., Siewertsen M., Enevold C., Hansen P.R., Kaur-Knudsen D., Zachariae C., Nielsen C.H., Loft N., Skov L. (2023). Blood Cell Biomarkers of Inflammation and Cytokine Levels as Predictors of Response to Biologics in Patients with Psoriasis. Int. J. Mol. Sci..

[16] Alves N.R.M., Kurizky P.S., da Mota L.M.H., de Albuquerque C.P., Esper J.T., Campos A.S.C., Reis V.P., Ferro H.M., Gil-Jaramillo N., Brito-de-Sousa J.P. (2024). Elevated serum IL-6 levels predict treatment interruption in patients with moderate to severe psoriasis: A 6-year real-world cohort study. An. Bras. Dermatol..

[17] Loft N.D., Skov L., Iversen L., Gniadecki R., Dam T.N., Brandslund I., Hoffmann H.J., Andersen M.R., Dessau R.B., Bergmann A.C. (2018). Associations between functional polymorphisms and response to biological treatment in Danish patients with psoriasis. Pharmacogenomics J..

[18] Butrón-Bris B., Llamas-Velasco M., Armesto S., Sahuquillo-Torralba A., Pujol-Montcusí J., Ruiz-Villaverde R., Martínez-López A., de la Cueva P., Romero-Maté A., Roustan G. (2025). Genetic Polymorphisms in Psoriasis: Investigating Genetic Variations for Precise Profiling of Response to Brodalumab in Real-Life Clinical Practice. Actas Dermo-Sifiliográficas.

[19] Andersch-Björkman Y., Micu E., Seifert O., Lonne-Rahm S.B., Gillstedt M., Osmancevic A. (2023). Effects of brodalumab on psoriasis and depressive symptoms in patients with insufficient response to TNF-α inhibitors. J. Dermatol..

[20] U.S. Food and Drug Administration (2015). Clinical Pharmacology and Biopharmaceutics Review: Brodalumab (Siliq).

[21] Krueger J.G., Morita A., Uchida-Yamada M., Tateishi C., Ogawa E., Masuda K., Yamaguchi Y., Hur H.B., Garcet S., Shishido-Takahashi N. (2025). Molecular profile of interleukin-17RA blockade by brodalumab in Japanese patients with psoriasis: Results from the ESPRIT study. J. Dermatol. Sci..

[22] Navrazhina K., Frew J.W., Grand D., Williams S.C., Hur H., Gonzalez J., Garcet S., Krueger J.G. (2022). Interleukin-17RA blockade by brodalumab decreases inflammatory pathways in hidradenitis suppurativa skin and serum. Br. J. Dermatol..

[23] Goldburg S.R., Strober B.E., Payette M.J. (2020). Hidradenitis suppurativa: Epidemiology, clinical presentation, and pathogenesis. J. Am. Acad. Dermatol..

[24] Liu S., He M., Jiang J., Duan X., Chai B., Zhang J., Tao Q., Chen H. (2024). Triggers for the onset and recurrence of psoriasis: A review and update. Cell Commun. Signal.

